# Hand Eczema in Apprentice Nurses during the COVID-19 Pandemic after a Skin Prevention Program

**DOI:** 10.3390/ijerph20042992

**Published:** 2023-02-08

**Authors:** Linda Piapan, Davide Di Taranto, Emilia Patriarca, Francesca Rui, Francesca Larese Filon

**Affiliations:** Unità Clinico Operativa di Medicina del Lavoro, Università di Trieste, Via della Pietà 2/2, 342129 Trieste, Italy

**Keywords:** hand eczema, COVID-19, apprentice nurses, hand hygiene regimen

## Abstract

Background: Healthcare workers, particularly nurses and apprentice nurses, are at high risk of the development of hand eczema due to daily exposure to wet work. This study aimed to assess the occurrence of hand eczema in a group of first-, second-, and third-year apprentice nurses at the University Hospitals of Trieste (northeastern Italy) during the COVID-19 pandemic. Methods: Two hundred forty-two Nursing School students were recruited. Data were collected using a standardized questionnaire based on the Nordic Occupational Skin Questionnaire, and all patients underwent a medical examination to evaluate their skin condition based on standard scores. Transepidermal water loss was also measured. The factors associated with hand eczema were investigated using univariate and multivariate logistic regression analyses. Results: The prevalence of hand eczema was low in students both before and after the traineeship (17.9 and 21.5%, respectively), but clinical signs of mild skin damage, mainly skin dryness, were present in 52.3 and 47.2%, respectively. The factor associated with hand eczema was a personal history of atopic eczema (odd ratios 2.61, 95% confidence intervals 1.18–5.80), while exposure to irritants and glove use did not reach statistical significance. Conclusions: Our findings might be explained by the preventive measures adopted for skin protection among healthcare workers in Trieste since the apprenticeship.

## 1. Introduction

Occupational skin diseases (OSDs) are one of the most common work-related diseases worldwide and considerably affect the quality of life and workability [1,2]. Hand eczema (HE) is the most common OSD and is responsible for up to 95% of all cases [1,3].

Healthcare workers, particularly nurses, are known to be at high risk of the development of OSDs due to daily exposure to wet work (frequent hand washing and prolonged glove wearing) in combination with chemicals [4,5]. The onset of OSDs occurs more often in apprentices than in qualified nurses [5,6] and often occurs in the first year of apprenticeship [7].

Nurse job tasks involve intense contact with water, soaps, detergents, occlusive gloves, disinfectants, and certain medical substances, all of which are well-known causes of irritant contact dermatitis (ICD) [7,8]. Additionally, nurses are exposed to allergens, such as rubber additives and natural latex glove proteins, preservatives, disinfectants (formaldehyde, glutaraldehyde), and drugs that can cause allergic contact dermatitis (ACD) [3,9]. Furthermore, irritant skin damage has been shown to be a risk factor for developing ACD [1].

This study aimed to assess the occurrence of HE in a group of first-year apprentice nurses before the start of training and in a group of second- and third-year apprentice nurses after training at the University Hospitals of Trieste (northeastern Italy) and establish the risk factors.

In addition, this study aimed to evaluate the impact of increased hygiene measures and the wearing of personal protective equipment (PPE) on skin damage during the COVID-19 pandemic.

## 2. Materials and Methods

An observational study was conducted with the aim of investigating the prevalence of HE in apprentice nurses in relation to their work experience and number of hours of training. Furthermore, we aimed to analyze the impact of the COVID-19 pandemic on skin barrier integrity.

### 2.1. Study Population

Students were recruited from the Nursing School of the University of Trieste, northeastern Italy. Students in the first, second, and third years were enrolled. The total population consisted of 242 students aged ≥19 years. Data from the first year were collected in March 2022 before the start of the traineeships in the hospital, and data from the second and third years were collected after the traineeships in the hospital in October 2021 and January 2022, respectively.

### 2.2. Assessment

Data were collected inside the classroom of the Department of Nursing School in Valmaura, Trieste, in accordance with the COVID-19 preventive measures. During data collection, enrolled students attended a one-hour class on OSDs and related preventive measures. All students had already attended a pre-traineeship course on preventive measures for occupational injuries and diseases lasting longer than 12 h. The purpose of the study was explained to all participants before beginning, and a specific form was signed to document the understanding and acceptance of the participants. Data were collected using a standardized questionnaire based on the Nordic Occupational Skin Questionnaire (NOSQ-2002) [10]. Participants had to fill out a form with their personal information: name, surname, age, sex, work, and the number of years they had worked. The multiple-choice questionnaire investigated the history of HE, the substances that worsened HE during the traineeship, the substances that worsened hand eczema in daily life, the improvement of HE out of work, the loss of a previous job because of HE, the history of atopic eczema during childhood, glove use during traineeship or work, gloves used in everyday life, symptoms related to glove use, number of hand washing during a shift, number of hours spent with wet hands, history of allergies and, if yes, drugs taken, family history of allergies, smoking habits, skin condition during the COVID-19 pandemic, and symptoms related to mask use. Irritant exposure was assessed using the questionnaire suggested by Uter et al. [11] and was reported as the sum of different irritant factors.

The participants were examined by a medical doctor who evaluated the condition of their skin by analyzing their fingertips, fingers, palm, back of the hand, and wrists. To quantify the results numerically, the Hand Eczema Severity Index (HECSI) [12] was used.

Skin damage was further examined by analyzing transepidermal water loss (TEWL) [13] in 73 students from both subgroups using a VapoMeter device (Delfin Technologies, Kuopio, Finland), and the results were expressed as g/m^2^/h. Two independent measures were taken on the first interdigital space between the thumb and index finger. To avoid altering the results, students were advised not to apply moisturizers or gels on their skin before the measurements.

### 2.3. Statistical Analysis

The results are reported using an Excel datasheet. Data were analyzed using both descriptive and logistic statistics in STATA version 14 (StataCorp, College Station, TX, USA).

Descriptive statistics were performed to compare the two groups, 1st year versus 2nd and 3rd years as the combined group. Age, interdigital TEWL, HECSI score, and hazard exposure were reported as the median (25–75° percentiles), and the difference between the two groups was analyzed using the Mann–Whitney test, as the data were not normally distributed. Categorical variables were reported in contingency tables as numbers and percentages, and the differences between the two groups were analyzed using the chi-square test.

Factors associated with HE (age, sex, childhood eczema, hours per day of glove use, number of hand washings per day, wet time per day, allergy history, smoking, interdigital TEWL, and hazard exposures) were processed using a logistic regression model, with univariable logistic regression. Significantly associated factors were then evaluated using a multivariate logistic regression and reported as odds ratios (ORs) and 95% confidence intervals (CI). A *P* significance was set at *p* < 0.05.

The Friuli Venezia Giulia Ethical Committee approved the study (CEUR No. 092/2018).

## 3. Results

### 3.1. Characteristics of the Study Population

The characteristics of the study population are summarized in Table 1. We divided the students into two subgroups: in the first group, only first-year apprentice nurses were included before the start of the traineeship (84 individuals, 60.9% eligible); in the second group, second- and third-year apprentice nurses who had already started the traineeship in hospitals were grouped together (158 individuals, 78.6% eligible). No differences in sex or age distribution were found between the students who participated in the study and those who did not.

A significant difference was observed in the median age between the two groups (20 vs. 21 years in the first and second group, respectively; *p* < 0.001), according to the course year. Women were the majority in both groups (80.7% vs. 79.7%, respectively), as expected, considering that women represent 75–80% of the healthcare workforce.

A history of HE was present in 17.9 and 21.5% of the first- and second–third-year groups, respectively (*p* = 0.500), and atopic eczema in childhood was reported in similar percentages in the two groups (11.9% vs. 13.9%). The smoking rate was higher in the second–third-year group (27.2%) than in the first-year group (18.3%).

**Figure 1:**
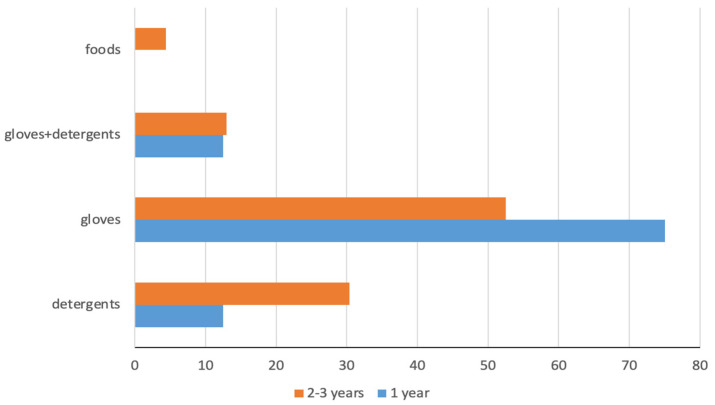
Substances that worsened HE.

**Figure 2:**
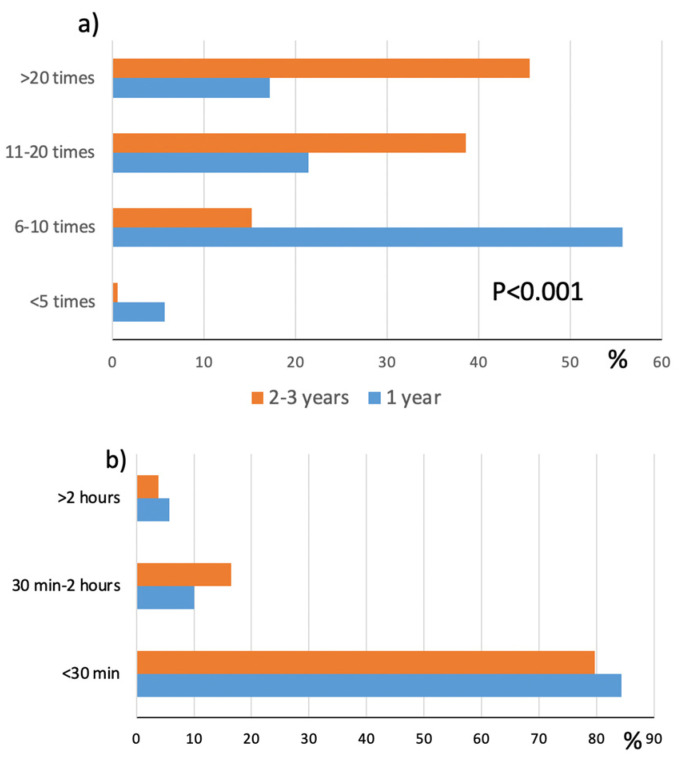
Distribution of hand washing per day (**a**) and wet time (**b**) in 1st-year and 2nd–3rd–year students.

In the first and second groups, 57 and 67% of those who had a history of eczema reported a worsening of HE with work, mainly in relation to glove use (75 and 52.5%, respectively) or by contact with detergents (Figure 1). The impairment of the skin barrier integrity was aggravated by the high number of hand washes performed by apprentice nurses during their shift, with significant differences between the two groups. The majority of first-year students washed their hands 6–10 times per day, while the majority of second–third years washed their hands more than 20 times per day (Figure 2a). However, only a small number of participants reported a wet time of more than 30 min (Figure 2b), indicating an awareness of the risk of wet work and suggested preventive measures.

**Figure 3:**
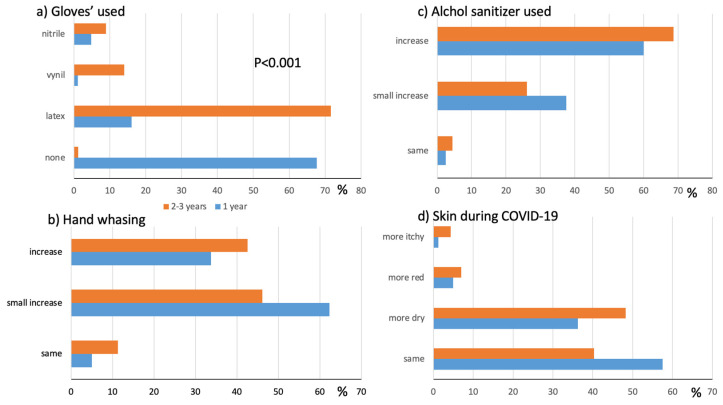
Impact of COVID-19 pandemic. Glove preferences (**a**), modification of hygiene habits (**b**), use of alcohol-based sanitizers (**c**), perception of own skin condition (**d**) in 1st-year and 2nd–3rd- year students.

The number of individuals who wore gloves during the day was significantly higher in the second group than in the first (45.2% vs. 17.5%, respectively; *p* < 0.001). Figure 3a shows the students’ glove preferences: the majority preferred the use of latex gloves, and those who answered ‘none’ to the question were all first-year students who had not yet started training. Glove-related symptoms, mostly itching and discomfort, were reported by 9.8% of the first group and 17.4% of the second group. However, five reported urticaria, and one reported rhinitis and asthma wearing gloves: all underwent skin prick tests with latex extracts that resulted in being negative, but latex gloves were forbidden for all of them.

In the clinical evaluation, dryness and desquamation of the palm and back of the hands were the most important skin changes found, while papules, vesicles, and cracking were found in very low numbers (two students in the first group and two students in the second group). No significant difference was observed in the percentage of positive clinical examinations in the first-year apprentice nurses (52.3%) vs. second–third-year apprentice nurses (46.2%). The median HECSI score, which assesses the intensity and severity of skin changes, was 1 in the first group and 0 in the second group, with a statistically significant difference (*p* = 0.007). Irritant exposure assessed using the Uter [11] questionnaire showed significant differences between the two groups, with a median value of 6 (IQR 0–12.5) and 21 (15–29) in the first-year and second–third-year students, respectively (*p* < 0.001). The IQR of the first group underlines the fact that first-year students had not started their training. Transepidermal water loss (TEWL) was assessed in 73 subjects; however, no significant difference was found between the two subgroups (*p* = 0.968).

The COVID-19 pandemic affected the habits of the apprentice nurses (Figure 3b–d): hand washing increased in both groups, in a smaller amount for the first group and more in the second–third-year group. The use of alcohol-based sanitizers increased significantly in both groups, and the perception of skin damage during the pandemic was altered in 42.5 and 60% of the first and second groups, respectively.

### 3.2. Factors Associated with Hand Eczema

The HE-associated factors were analyzed using univariate and multivariate logistic regressions (Table 2). Childhood eczema was the principal factor that predicted HE (OR 2.61, 95% CI 1.18–5.80). Statistical significance was almost achieved when considering the number of hours per day of glove usage.

## 4. Discussion

Our study assessed the prevalence and risk factors of HE in apprentice nurses from vocational schools at Trieste University Hospitals (northeastern Italy) during the COVID-19 pandemic before and after training. The prevalence of hand eczema was slightly higher after the traineeship, but the difference did not reach statistical significance (17.9 and 21.5% in first vs. second–third groups, respectively, *p* = 0.500). The presence of ongoing HE was reported in the same percentage of the two groups considered (3.6% vs. 3.2% before and after the traineeship). This prevalence is very low compared with other similar data among apprentice nurses. A recent study by Sakic et al. (2022) [9] analyzed a group of apprentice nurses after the traineeship and found a prevalence of HE of 46%, but their study population was characterized by fifth-year students and a double prevalence of atopic eczema in childhood, which is one of the most important risk factors for HE. 

It is known from the literature that the tendency to develop HE increases with the duration of exposure to risk factors at work [9,14] and can occur within a few years of practical work [7,15]. However, we did not find a significant difference between the exposed and non-exposed students. Although not significant, the prevalence of apprentice nurses complaining about symptoms related to the use of gloves was higher in the second–third-year group compared to the first-year group (17.4% vs. 9.8%), probably due to the higher exposure to glove usage by the second–third-year apprentices during practical training in vocational school. However, the vast majority complained of non-specific glove-related symptoms, such as discomfort and itching, while the prevalence of glove-related eczema was only about 1% in both groups. Filon et al. observed a decrease in glove-related symptoms among HCWs from 2000 to 2009 due to the introduction of non-powdered latex gloves for all workers and non-latex gloves for symptomatic or sensitized individuals in Trieste University Hospitals [16]. 

On clinical examination of the hands, no statistically significant differences were found between the two groups; 44 (52.4%) first-year students and 73 (46.2%) of second–third-year students had clinically observed skin changes, mostly dryness, on examination. Although dry skin is a known risk factor for dermatitis [17,18,19], the majority of our apprentices had no other clinical signs of HE. 

Regarding the severity of HE, the median HECSI was 1 (IQR 0–2) and 0 (IQR 0–1) in the first and second groups, respectively, implying that the skin had no or mild changes. In contrast to our findings, half of the apprentice nurses in the study of šakić et al. during the COVID-19 pandemic had clinically observed HE, with skin mostly affected by erythema, infiltration, and desquamation of mild to moderate severity [9].

In addition to clinical evaluation, we chose to use TEWL measurements as a tool to evaluate the compromised barrier function in apparently healthy skin, as it is widely accepted by several authors [13,15,20]_._ In both apprentice groups, the observed TEWL measurements were similar. Overall, our apprentice nurses had low skin susceptibility to eczema. However, our results differ from those of other studies in several countries in which apprentices are still at increased risk for OSDs, with an incidence more than 100 times higher than that in cohorts of trained HCWs [5]. 

The World Health Organization and Centers for Disease Control and Prevention have repeatedly stressed the importance of preventive behaviors during the COVID-19 pandemic, such as washing hands with soaps or using sanitizers more frequently. Simultaneously, increased skin disinfection practices and prolonged usage of PPE have been reported as risk factors for the development of cutaneous reactions [4]. According to health regulations, most apprentices studied (91%) reported changes in daily hygiene habits during the pandemic, and almost all (96%) reported mild-to-severe differences in their disinfecting routine with alcohol-based sanitizers. Nevertheless, approximately half of the participants (52%) felt skin changes, mostly dry and chapped hands, related to COVID-19 hygiene practices, and only a few complained of flushing and itching. Dry skin has already been reported as the most frequent skin change observed in HCWs during the pandemic, but the rates of cutaneous reactions to pandemic preventive hygiene measures reported in the literature have been much higher, affecting up to 97% of HCWs [21,22,23,24,25,26,27].

The lack of consistent self-reported and clinically observed skin changes in our study on apprentices could indicate a certain degree of the impact of preventive efforts implemented in Trieste University Hospitals since the outbreak of latex allergy more than 20 years ago [16,28,29]. Such efforts include specific training and classrooms that regularly emphasize skin protection among healthcare personnel. All workers and apprentices were recommended to (1) use appropriate PPE, preferring those with less irritant and sensitizing potential and/or combined with cotton undergloves; (2) use alcohol-based sanitizers as less irritant agents than classical washing with water and soap; and (3) to combine gentle cleaning that respects the skin pH with the use of emollient creams to reduce skin damage induced by wet work. When the COVID-19 pandemic started, all HCWs and apprentices had to use alcohol-based sanitizers to reduce their use of soaps. The effectiveness of the adopted preventive measures has also been evaluated in HCWs in our hospitals [30]. 

Finally, factors related to HE were studied using both univariate and multivariate analyses. A history of childhood eczema was the only factor significantly and positively associated with HE (OR 2.61, 95% CI 1.18–5.80). This finding confirms that childhood eczema is an important endogenous risk factor for HE in adulthood [31]. However, we did not find an association with personal factors, such as sex and age. Conversely, the literature data report that women, apprentices, and young workers are at increased risk of developing HE [6]. A distortion regarding sex could be present because approximately 80% of the study participants were female. Moreover, we did not observe an association between smoking and HE, in contrast to other studies that have recognized smoking as a behavioral risk factor, which is also associated with the severity or poor prognosis of eczema itself [32,33]. However, it is possible that our study lacked the power to detect potential associations due to the small number of apprentices with eczema.

Our study has some limitations related to the small sample size recruited from a single center in Trieste, northeastern Italy. Another limitation is the lack of information about the frequency of use of emollient creams suggested during traineeship. The strengths of our study include the analysis of apprentice nurses for all three years of vocational school in Trieste University Hospitals using a standardized questionnaire, a clinical evaluation performed by medical doctors, and the measurement of TEWL.

## 5. Conclusions

Altogether, our findings suggest a lower prevalence and severity of clinically observed and self-reported HE in apprentice nurses at the University of Trieste during the COVID-19 pandemic and no association with work-related habits (duration of wet work, frequency of hand washing, and glove usage). No significant difference was found between the first and second–third-year apprentices in terms of skin conditions (also evaluated using TEWL measurements). Such results might be due to a general low skin susceptibility to develop eczema in our apprentices with a low prevalence of atopic eczema in childhood but also to the importance given to skin protection among our HCWs since the apprenticeship. Over the three years of the vocational education program, 12 h were dedicated to information and training to increase apprentices’ awareness of occupational exposure and proper preventive measures, emphasizing health regulations on hand hygiene. In particular, alcohol-based sanitizers with handwashing with water and soap were recommended, and glove materials and immunogenic capacities were explained. Furthermore, during the first medical visit, which every student undertook before beginning the traineeship, subjects at higher risk (subjects with family or personal atopy, a skin prick test positive for common allergens, or contact dermatitis) were identified, and an occupational physician further highlighted such recommendations. Moreover, before the beginning of our study, all recruited students took a one-hour class in which an occupational physician educated them about hand hygiene, demonstrating how hand washing and disinfection should be performed, emphasizing the importance of appropriate moisturizer use. In this context of prevention activities in Trieste University Hospitals, pandemic hygiene regimens would seem to have had little impact on skin barrier integrity among our apprentices. 

## Figures and Tables

**Table 1:** Characteristics of apprentice nurses studied. In bold are reported significant values.

Characteristics	1st-Year Apprentices before Traineeship (No. = 84)	2nd–3rd-Year Apprentices (No. = 158)	*p* Value
Age (years), median (IQR)	**20 (19–23)**	**21 (21–24)**	**<0.001**
Women, No. (%)	72 (80.7)	126 (79.7)	0.252
- Hand eczema No. (%)	15 (17.9)	34 (21.5)	0.500
- Symptoms ongoing	3 (3.6)	5 (3.2)	
- Symptoms in the last 3 months	2 (2.4)	10 (6.3)	
- Symptoms in the last 3–12 months	4 (4.8)	11 (7.0)	
- Symptoms more than a year ago	6 (7.1)	8 (5.1)	
Worsening hand eczema during job, No. (%)	8 (57.1)	23 (67.65)	0.263
Childhood eczema, No. (%)	10 (11.9)	22 (13.9)	0.659
Use of gloves during the day, No. (%)	**7 (17.5)**	**71 (45.2)**	**0.001**
Pairs of gloves used per shift, median (IQR)	17.5 (10–50)	20 (20–30)	0.563
Hours wearing gloves, median (IQR)	5 (4–6)	6 (4–7)	0.599
Symptoms related to glove use, No. (%)	8 (9.8)	27 (17.4)	0.114
- Non-specific symptoms (i.e., itching and discomfort)	7 (8.3)	23 (14.6)	0.162
- Eczema	1 (1.2)	2 (1.3)	0.960
- Urticaria	1 (1.2)	4 (2.5)	0.485
- Allergic rhinitis	0 (0)	1 (0.6)	-
- Asthma	0 (0)	1 (0.6)	-
Allergies, No. (%)	19 (22.6)	42 (26.6)	0.499
- Oculorhinitis	7 (8.3)	24 (15.2)	0.129
- Asthma	7 (8.3)	11 (7)	0.699
- Drugs taken	2 (2.4)	22 (13.9)	**0.004**
Family history of allergies, No. (%)			
- Respiratory symptoms	21 (25.3)	36 (22.8)	0.603
- Skin symptoms	10 (12)	26 (16.5)	0.452
Smokers, No. (%)	15 (18.3)	43 (27.2)	0.059
Interdigital TEWL, median (IQR), total sample: No. = 73	16 (13.7–20.7)	17.3 (12.5–25.6)	0.450
Positive clinical examination, No. (%)	44 (52.38)	73 (46.2)	0.360
HECSI score, median (IQR)	1 (0–2)	0 (0–1)	**0.007**
Exposure hazard, median (IQR)	6 (0–12.5)	21 (15–29)	**<0.001**

IQR: interquartile range; TEWL: trans-epidermal water loss.

**Table 2:** Factors associated with HE investigated using univariate and multivariate logistic regression analysis. Associations are reported as odds ratios (ORs) and 95% confidence interval (CI). Statistically significant data are highlighted in bold.

Factors	Univariable OR	95% CI	*p* Value	Multivariate OR	95% CI	*p* Value
Age	0.98	0.94–0	0.434	0.98	0.94–1.02	0.472
Sex	1.15	0.60–2.21	0.671	1.13	0.58–2.20	0.709
Hand eczema	1.55	0.82–2.93	0.169			
Childhood eczema	**2.66**	**1.20–5.89**	**0.016**	**2.61**	**1.18–5.80**	**0.018**
Glove use y/n	0.98	0.91–1	0.188			
Hours per day of glove use	1.15	0.99–1.32	0.055			
Symptoms after wearing gloves	1.16	0.57–2.39	0.670			
Number of hand washing per day	0.96	0.71–1.3	0.816			
Wet time	0.69	0.41–1.16	0.162			
Allergy	1.2	0.69–2.22	0.458			
Smoking	1.38	0.76–2.51	0.286			
Interdigital TEWL	0.99	0.95–1.04	0.968			
Hazard exposure	0.98	0.96–1	0.088			

TEWL: transepidermal water loss.

## Data Availability

Data will be available on request.

## References

[1] Diepgen T.L. (2012). Occupational skin diseases. J. Dtsch. Dermatol..

[2] Wiszniewska M., Walusiak-Skorupa J. (2015). Recent Trends in Occupational Contact Dermatitis. Curr. Allergy Asthma Rep..

[3] Lampel H.P., Powell H.B. (2019). Occupational and Hand Dermatitis: A Practical Approach. Clin. Rev. Allergy Immunol..

[4] Hamnerius N., Svedman C., Bergendorff O., Björk J., Bruze M., Pontén A. (2018). Wet work exposure and hand eczema among healthcare workers: A cross-sectional study. Br. J. Dermatol..

[5] Filon F.L., Pesce M., Paulo M.S., Loney T., Modenese A., John S.M., Kezic S., Macan J. (2021). Incidence of occupational contact dermatitis in healthcare workers: A systematic review. J. Eur. Acad. Dermatol. Venereol..

[6] Flyvholm M.A., Bach B., Rose M., Jepsen K.F. (2007). Self-reported hand eczema in a hospital population. Contact Dermat..

[7] Visser M.J., Verberk M.M., van Dijk F.J., Bakker J.G., Bos J.D., Kezic S. (2014). Wet work and hand eczema in apprentice nurses; part I of a prospective cohort study. Contact Dermat..

[8] Carøe T.K., Ebbehøj N., Agner T. (2014). A survey of exposures related to recognized occupational contact dermatitis in Denmark in 2010. Contact Dermat..

[9] Šakić F., Babić Ž., Franić Z., Macan J. (2021). Characteristics of hand eczema in final-year apprentice nurses during the COVID-19 pandemic. Contact Dermat..

[10] Susitaival P., Flyvholm M.-A., Meding B., Kanerva L., Lindberg M., Svensson A., Olafsson J.H. (2003). Nordic Occupational Skin Questionnaire (NOSQ- 2002): A new tool for surveying occupational skin diseases and exposure. Contact Dermat..

[11] Uter W., Bauer A., Bensefa-Colas L., Brans R., Crépy M.-N., Giménez-Arnau A., Filon F.L., Hadžavdić S.L., Pesonen M., Schuttelaar M.L. (2018). Extended documentation for hand dermatitis patients: Pilot study on irritant exposures. Contact Dermat..

[12] Oosterhaven J.A.F., Schuttelaar M.L.A. (2020). Responsiveness and interpretability of the Hand Eczema Severity Index. Br. J. Dermatol..

[13] Alexander H., Brown S., Danby S., Flohr C. (2018). Research Techniques Made Simple: Transepidermal Water Loss Measurement as a Research Tool. J. Invest. Dermatol..

[14] Franić Z., Babić Ž., Bjelajac A., Macan J. (2019). Factors related to skin health in hairdressing apprentices from two Croatian regions. Contact Dermat..

[15] Schmid K., Broding H.C., Uter W., Drexler H. (2005). Transepidermal water loss and incidence of hand dermatitis in a prospectively followed cohort of apprentice nurses. Contact Dermat..

[16] Larese Filon F., Bochdanovits L., Capuzzo C., Cerchi R., Rui F. (2014). Ten years incidence of natural rubber latex sensitization and symptoms in a prospective cohort of health care workers using non-powdered latex gloves 2000–2009. Int. Arch. Occup. Environ. Health.

[17] Aktas E., Esin M.N. (2016). Skin disease symptoms and related risk factors among young workers in high-risk jobs. Contact Dermat..

[18] Piapan L., Baldo J., Larese Filon F. (2019). Occupation-Related Symptoms in Hairdressers. Dermatitis.

[19] Smit H.A., van Rijssen A., Vandenbroucke J.P., Coenraads P.J. (1994). Susceptibility to and incidence of hand dermatitis in a cohort of apprentice hairdressers and nurses. Scand. J. Work Environ. Health.

[20] Fluhr J.W., Feingold K.R., Elias P.M. (2006). Transepidermal water loss reflects permeability barrier status: Validation in human and rodent in vivo and ex vivo models. Exp. Dermatol..

[21] Lin P., Zhu S., Huang Y., Li L., Tao J., Lei T., Song J., Liu D., Chen L., Shi Y. (2020). Adverse skin reactions among healthcare workers during the coronavirus disease 2019 outbreak: A survey in Wuhan and its surrounding regions. Br. J. Dermatol..

[22] Lan J., Song Z., Miao X., Li H., Li Y., Dong L., Yang J., An X., Zhang Y., Yang L. (2020). Skin damage among health care workers managing coronavirus disease-2019. J. Am. Acad. Dermatol..

[23] Yuan X., Xi H., Le Y., Xu H., Wang J., Meng X., Yang Y. (2021). Online survey on healthcare skin reactions for wearing medical-grade protective equipment against COVID-19 in Hubei Province, China. PLoS ONE.

[24] Alluhayyan O.B., Alshahri B.K., Farhat A.M., Alsugair S., Siddiqui J.J., Alghabawy K., AlQefari G.B., Alolayan W.O., Abu Hashem I.A. (2020). Occupational-Related Contact Dermatitis: Prevalence and Risk Factors Among Healthcare Workers in the Al’Qassim Region, Saudi Arabia During the COVID-19 Pandemic. Cureus.

[25] Erdem Y., Altunay I.K., Çerman A.A., Inal S., Ugurer E., Sivaz O., Kaya H.E., Gulsunay I.E., Sekerlisoy G., Vural O. (2020). The risk of hand eczema in healthcare workers during the COVID-19 pandemic: Do we need specific attention or prevention strategies?. Contact Dermat..

[26] Kiely L.F., Moloney E., O’Sullivan G., Eustace J.A., Gallagher J., Bourke J.F. (2021). Irritant contact dermatitis in healthcare workers as a result of the COVID-19 pandemic: A cross-sectional study. Clin. Exp. Dermatol..

[27] Guertler A., Moellhoff N., Schenck T.L., Hagen C.S., Kendziora B., Giunta R.E., French L.E., Reinholz M. (2020). Onset of occupational hand eczema among healthcare workers during the SARS-CoV-2 pandemic: Comparing a single surgical site with a COVID-19 intensive care unit. Contact Dermat..

[28] Larese Filon F., Bosco A., Fiorito A., Negro C., Barbina P. (2001). Latex symptoms and sensitisation in health care workers. Int. Arch. Occup. Environ. Health.

[29] Filon F.L., Radman G. (2006). Latex allergy: A follow up study of 1040 healthcare workers. Occup. Environ. Med..

[30] Piapan L., Bramuzzo D., Rui F., Filon F.L. (2022). Incidence of skin diseases in healthcare workers before and during the COVID-19 pandemic at Trieste hospitals (northeastern Italy). Contact Dermat..

[31] Johannisson A., Pontén A., Svensson Å. (2013). Prevalence, incidence and predictive factors for hand eczema in young adults—A follow-up study. BMC Dermatol..

[32] Olesen C.M., Agner T., Ebbehøj N.E., Carøe T.K. (2019). Factors influencing prognosis for occupational hand eczema: New trends. Br. J. Dermatol..

[33] Sørensen J.A., Fisker M.H., Agner T., Clemmensen K.K., Ebbehøj N.E. (2017). Associations between lifestyle factors and hand eczema severity: Are tobacco smoking, obesity and stress significantly linked to eczema severity?. Contact Dermat..

